# Acute *Propionibacterium acnes* Infection After Carpal Tunnel Release

**DOI:** 10.1016/j.jhsg.2021.06.008

**Published:** 2021-07-31

**Authors:** Jeffrey Bortman, Alan Schefer

**Affiliations:** ∗ New York Medical College School of Medicine, Westchester Medical Center, Valhalla, NY; † Department of Orthopedic Surgery, CareMount Medical, Mount Kisco, NY

**Keywords:** Carpal tunnel syndrome, Infection, *Propionibacterium acnes*

## Abstract

*Propionibacterium acnes* (*P. acnes*) is a gram-positive, anaerobic, commensal organism found on nonglabrous skin, including the face, scalp, chest, and axilla. Recently, *P. acnes* is one of the more frequently recognized organisms causing postoperative infections in implant surgery of the shoulder, second to only *Staphylococcus aureus* (*S. aureus*), yet it is a rare postoperative complication of the wrist and hand. Multiple factors, including slow growth, multiorganism involvement, and selective growth media, complicate attributing a primary infection to *P. acnes*. We present a case of primary, acute *P. acnes* infection after carpal tunnel decompression, demonstrating the need for considering *P. acnes* for persistent postoperative hand and wrist infections.

Carpal tunnel syndrome is the most common compression neuropathy in the United States, with more than 600,000 carpal tunnel release procedures annually.^1^ Carpal tunnel release relieves the symptoms of numbness in the sensory distribution of the median nerve, pain, and/or weakness of the thenar muscles. Procedure-related complications, including infection, scar tenderness, wrist stiffness, and nerve injury, are rare after an elective decompression surgery, but remain important postoperative considerations.^1^

*Propionibacterium acnes* (*P. acnes*) is a non–spore forming, gram-positive, gas-forming, anaerobic bacillus. It is a commensal organism of the skin and mucosal flora and is typically found on the face, chest, axilla, and thorax.^2^ *P. acnes* primarily infects the nutrient-rich, immunologically privileged synovial sites such as the shoulder, hip, and knee after surgery, and it is currently the second most common pathogenic organism following shoulder arthroplasty, behind only *Staphylococcus aureus* (*S. aureus*).^2^ Although well-known to shoulder surgeons because of *P. acnes*’ preferred habitat within hair follicles of the axilla, *P. acnes* infection has been rarely reported to cause an infection in the glabrous hand or wrist.^3^^,^^4^ To the best of our knowledge, we report the first case of acute *P. acnes* infection after open carpal tunnel decompression. We aim to broaden the awareness among hand surgeons for the potential for *P. acnes* infection following hand and wrist surgery and to recommend the creation of formal, evidence-based guidelines for the treatment of deep-tissue *P. acnes* infection.

## Case Description

### Initial presentation

A 49-year-old female patient with a past medical history notable for hypertension, type 2 diabetes mellitus (glycated hemoglobin = 7.8 at 5 days before surgery with normal fasting glucose), morbid obesity (body mass index = 42.8 kg/m^2^), chronic kidney disease, peripheral neuropathy, chronic obstructive pulmonary disease, and hypoalbuminemia presented to our institution with numbness and pain in her right and left hands. A physical examination was consistent with bilateral carpal tunnel syndrome and cubital tunnel syndrome, which was worse on the right side. Electrodiagnostic studies confirmed bilateral carpal and cubital tunnel syndromes.

The patient underwent a single, multisite operation, including right, open carpal tunnel release, followed by right decompression and anterior transposition of the ulnar nerve at the elbow under general anesthesia and supraclavicular nerve block. A preoperative dose of cefazolin (3 g) was administered as per the hospital protocol for all clean, elective surgeries, and the skin was prepared with Hibiclens Antiseptic Skin Cleanser (Hibiclens) and BD ChloraPrep (BD). The carpal tunnel was approached through a standard, 2.5–3-cm longitudinal incision in the palm, and the cubital tunnel was approached via a curved longitudinal incision posterior to the medial epicondyle. The ulnar nerve was subcutaneously transposed after decompression. Surgery was uneventful, and the patient was discharged with sterile dressings and a long-arm cast to protect the cubital tunnel incision. Postoperative wound care instructions included the removal of the sterile soft bandage on the hand on day 4 after surgery. The hand wound was to be cleaned with soap and water, dried well, and then covered with a large Band-Aid, which was to be changed daily or every time the hand was washed. The patient was scheduled for a follow-up appointment 2 weeks after surgery for removal of the long-arm cast and the sutures from the elbow and hand.

### Postsurgical complications

**Figure 1:**
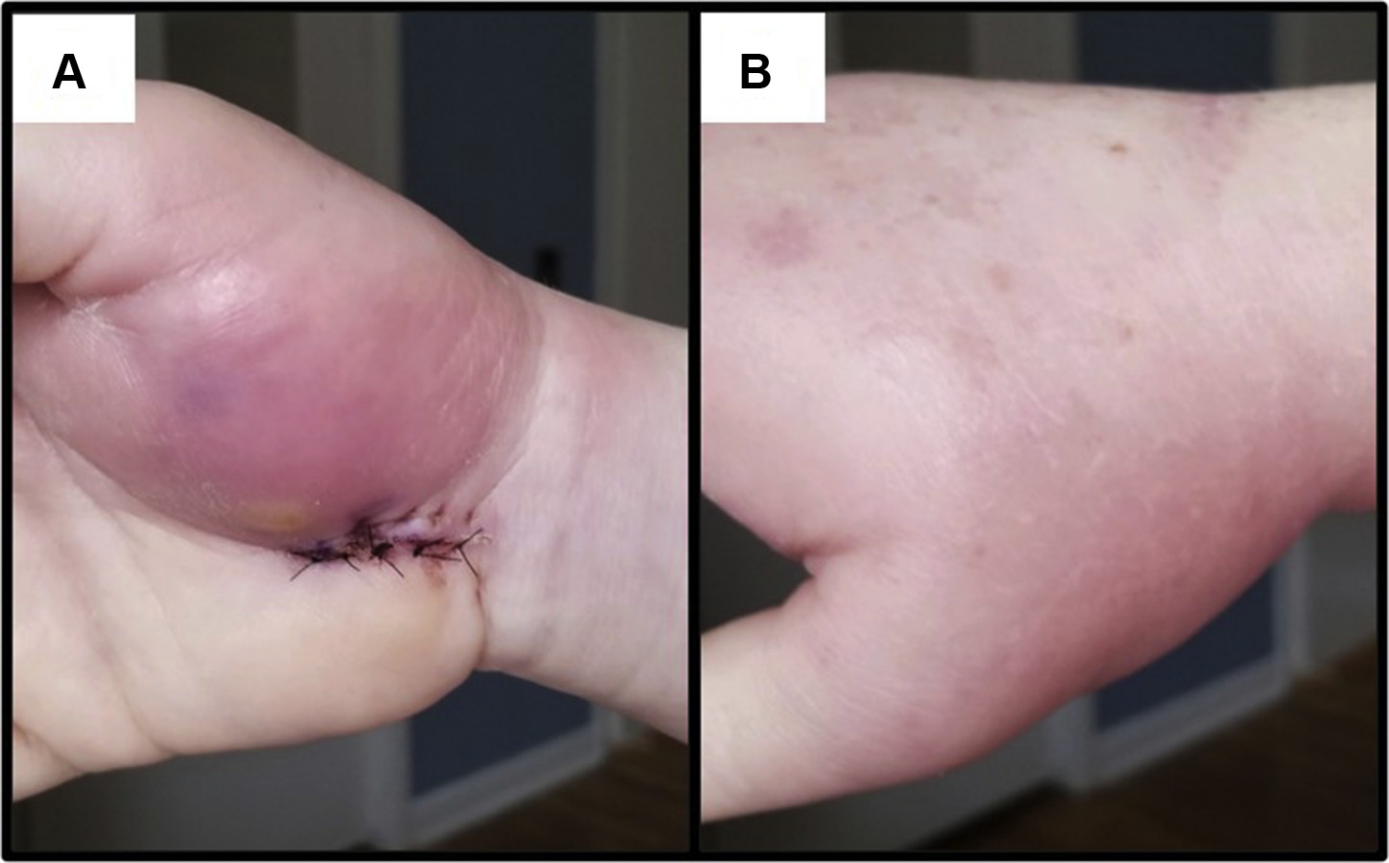
Right hand at 11 days after surgery. **A** Palmar aspect. **B** Dorsal aspect.

Eleven days after surgery, the patient was evaluated for increasing redness, pain, and swelling in her right hand over the right thenar eminence and carpal tunnel, including ascending lymphangitis and cellulitis of the region (Fig. 1). The elbow wound was well healed without evidence of infection, and the patient was afebrile. The patient was admitted for a deep-space infection with possible compartment syndrome to the thenar compartment and was scheduled for emergent incision and drainage (I&D), debridement, and fasciotomy. The I&D revealed cloudy serous fluid down to the deep carpal tunnel space and purulent tenosynovitis around the flexor tendons. A tenosynovectomy was performed around the affected tendons. A subsequent fasciotomy of the thenar compartment was performed. All other compartments remained soft at this time. Cultures of the deep spaces and tenosynovium were sent to the laboratory. The patient was then placed on broad-spectrum antibiotics consisting of vancomycin and pipercillin-tazobactam.

**Figure 2:**
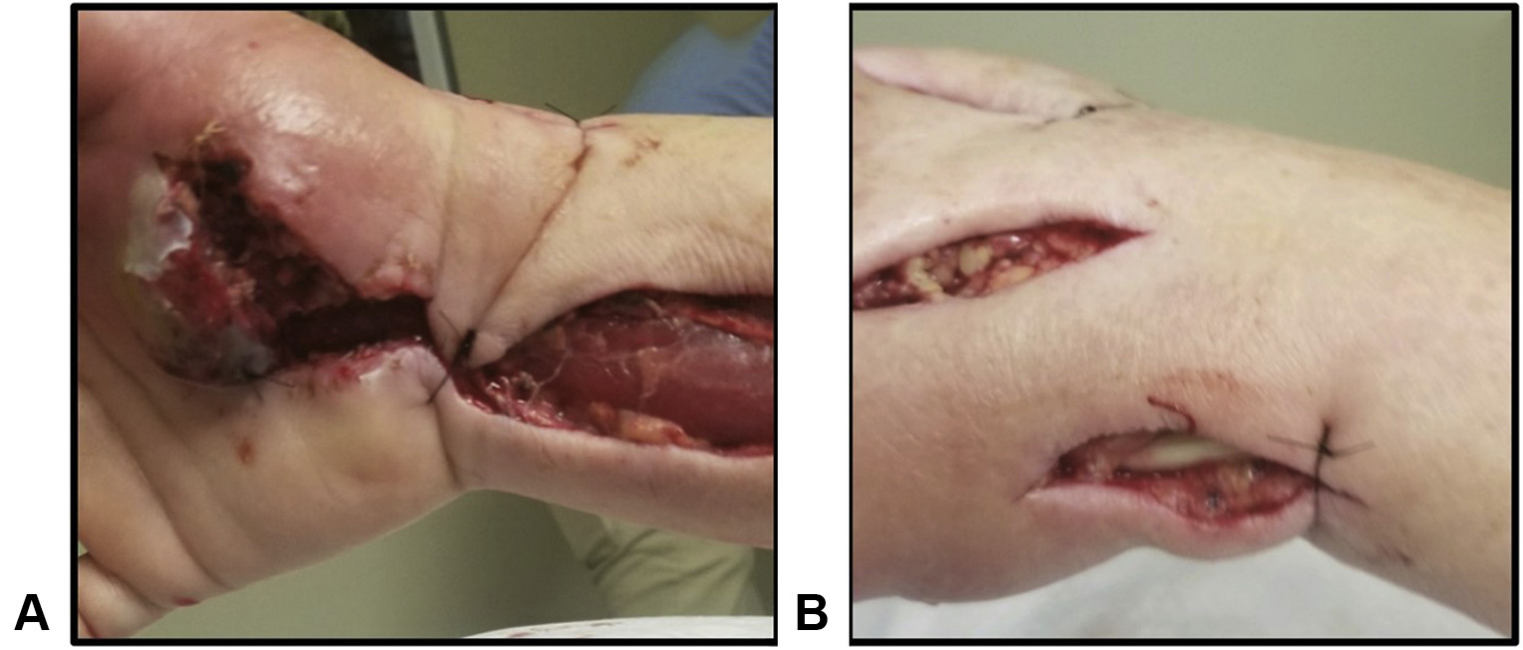
Hand wounds after second I&D and fasciotomy. **A** Palmar aspect of the hand, showing carpal tunnel and volar forearm debridement. **B** Dorsal aspect of the hand, showing compartment releases.

Despite extensive I&D and antibiotics, the infection continued to worsen. Less than 24 hours after surgery, the patient had worsening of pain and swelling. The clinical examination was worrisome now for compartment syndrome of the volar forearm compartment and remaining compartments of the hand. She went back to the operating room for a fasciotomy of the affected compartments and radical flexor tenosynovectomy (Fig. 2). The patient went back to the operating room 1 more time for I&D 9 days after admission because of sudden increases in swelling, erythema, and pain and a high white blood cell count. Reaccumulation of murky fluid was observed in the carpal tunnel despite previous drain placement, and there was skin necrosis over the thenar musculature. In the setting of obvious clinical disease progression, the decision was made to discontinue the patient’s intravenous pipercillin-tazobactam and switch to meropenem.

### Laboratory findings and imaging

**Figure 3:**
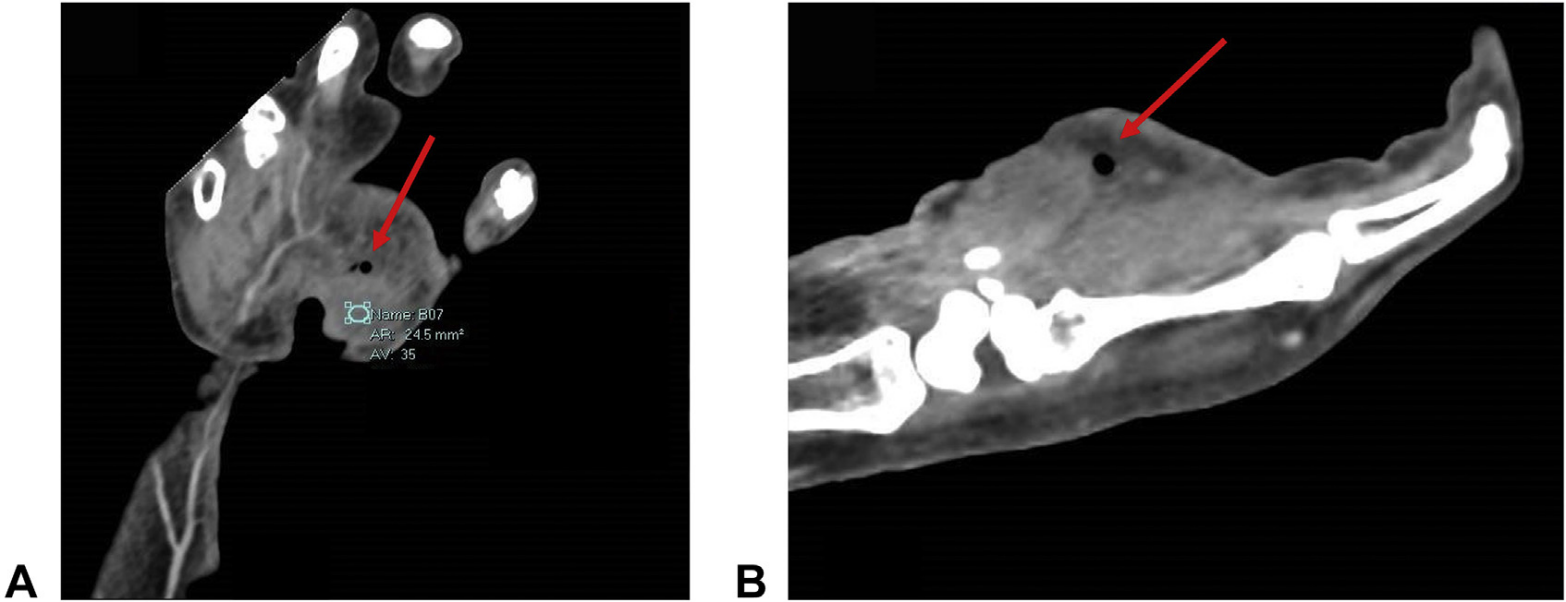
X-ray revealing phlegmon and gas formation in the distal thenar eminence (red arrows). **A** Coronal image. **B** Sagittal imagef.

A wound culture grew coagulase-negative *Staphylococcus* and *P. acnes* on the sixth day after surgery. Laboratory findings were notable for a persistently elevated white blood cell count (WBC) (12,910–22,580 WBCs/μL) throughout the infection. A computed tomography scan of the right upper extremity with contrast was performed prior to the third debridement to rule out fluid collection or necrotizing fasciitis. Findings revealed changes related to the previous surgical intervention and the possibility of phlegmon and gas formation in the distal thenar eminence (Fig. 3). Plain hand radiographs showed no evidence of osteomyelitis.

### Recovery

**Figure 4:**
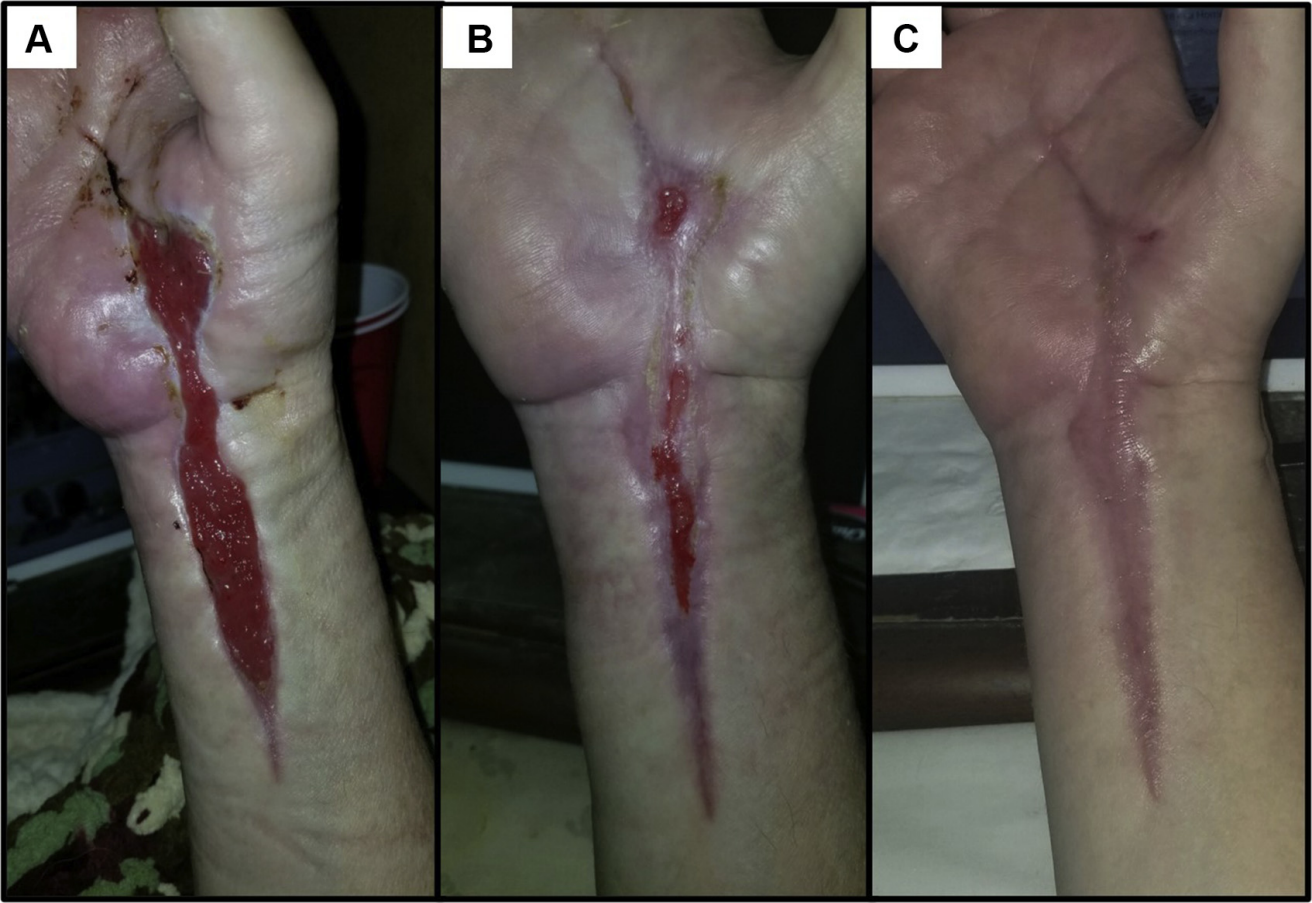
Healing process of the palmar aspect of the hand and wrist after initial I&D. **A** At 3.5 weeks. **B** At 6.5 weeks. **C** At 8.5 weeks.

**Figure 5:**
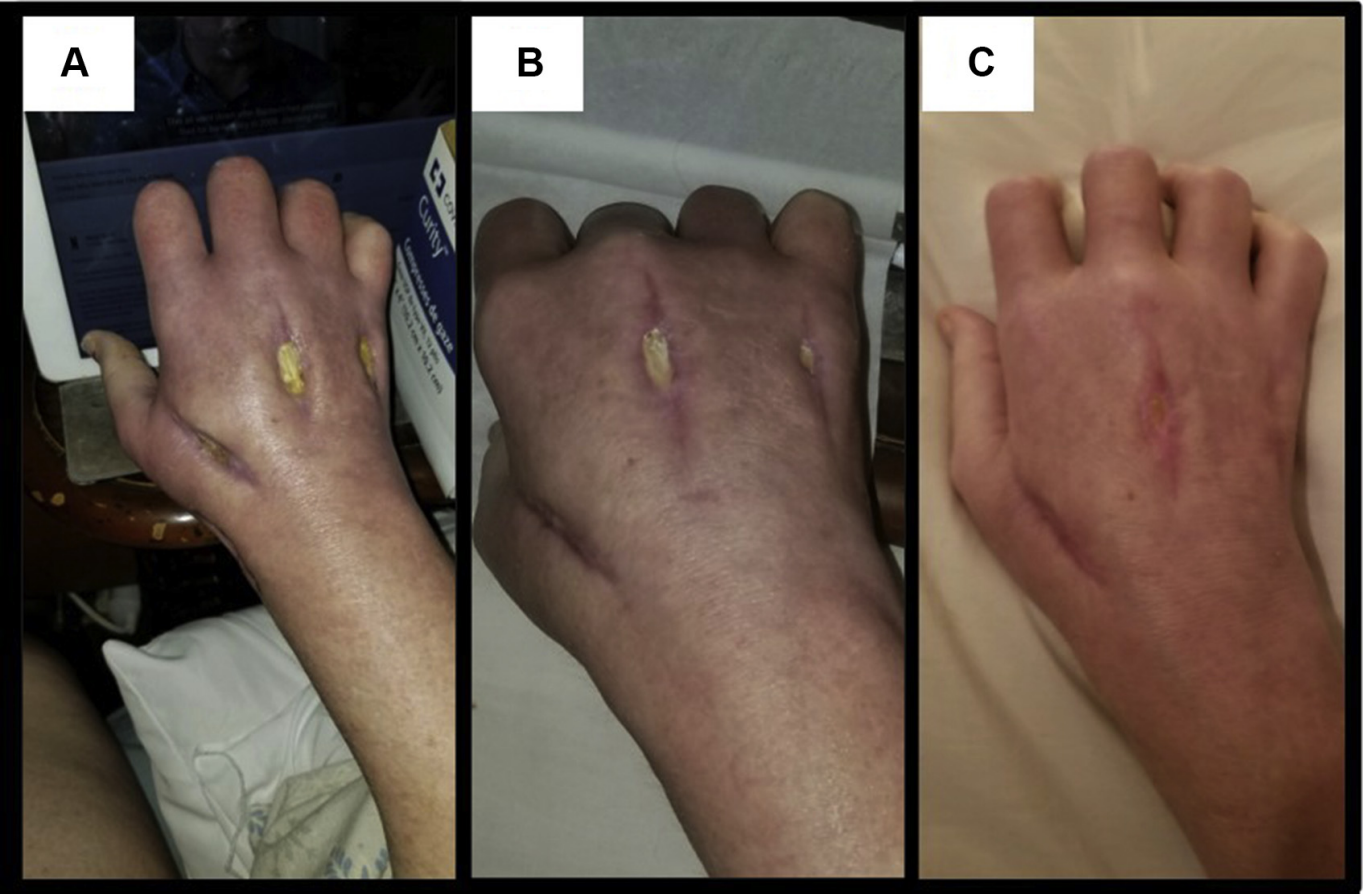
Healing process of the dorsal aspect of the hand after initial I&D. **A** At 4.5 weeks. **B** At 7.5 weeks. **C** At 11 weeks.

After the third debridement and antibiotic change, the patient made steady improvement with granulating hand wounds, decreasing leukocytosis, and resolving infection until she was stable to be discharged on day 22 of this admission, which was 32 days after her initial procedure. She was transferred to a skilled nursing facility to continue intravenous antibiotics and wound care. Discharge medications included intravenous vancomycin and meropenem via a peripherally inserted central catheter line and oral oxycontin, dilaudid, and pregabalin for pain. Despite leaving her skilled nursing facility after 12 hours and stopping all antibiotics against medical advice, the patient’s infection resolved, and her wounds healed over the course of 3 months (Figs. 4, 5). The patient was seen intermittently for follow-up and debridement of her healing wounds, and she demonstrated resolution of numbness, pain, and tingling in both the median and ulnar nerve distributions, suggesting successful nerve releases.

## Discussion

*S. aureus* and coagulase-negative *Staphylococcus* are commensal pathogens known to frequently complicate prosthetic and nonprosthetic procedures and are thus empirically treated for based on their prevalence. However, in antibiotic-refractory cases that continue to grow only *Staphylococcal* species, *P. acnes* must be suspected. It is known that *P. acnes* is a slow-growing organism that resides in the sebaceous glands, making it resistant to routine surface sterilization and allowing it to seed surgical sites during an incision.^5^ Because of this, *P. acnes* is much more prevalent in regions with a high density of sebaceous glands, such as the scalp, face, chest, and shoulder, and is rare in the areas of low sebaceous gland density, such as the wrist and hand. As such, *P. acnes* may be underreported in instances of postsurgical hand and wrist infection.

In this case, *P. acnes* was suspected as the primary pathogenic organism despite the growth of coagulase-negative *Staphylococcus* because of the surgery type, antibiotic sensitivity, computed tomography findings, and infection location. The consulted infectious disease specialist attributed the extent of the infection to *P. acnes* invasion, stating that coagulase-negative *Staphylococcus* was an unlikely primary pathogen because there was no surgical hardware involved in the procedure. It has been hypothesized that coagulase-negative *Staphylococcus* is particularly virulent with involvement of surgical hardware because of its ability to form biofilms and to invade and survive within phagocytes, such as osteoblasts.^6^ Furthermore, the patient’s prolonged, refractory antibiotic course was also suspicious for a *P. acnes* primary infection in the setting of antibiotic pan-sensitive *Staphylococcus*, especially considering empiric treatment with vancomycin and subsequent confirmation of vancomycin sensitivity by the microbiology laboratory. Additionally, computed tomography with contrast confirmed the presence of a gas-producing organism, which further suggested a *P. acnes* primary infection.^7^ Finding *P. acnes* in the glabrous region of the palm suggested extensive *P. acnes* proliferation and invasion from a sebum-producing (nonglabrous) region, such as the pores and hair follicles of the wrist or dorsal hand during surgery; however, it is unlikely that the consecutive, multisite surgery contributed to seeding of the wrist infection because the carpal tunnel release was completed before the cubital tunnel release.

Although the exact cause of such an extensive *P. acnes* infection is unknown, patient comorbidities, including type 2 diabetes mellitus, hypoalbuminemia, and obesity, likely contributed to the severity of infection. Diabetes considerably increases morbidity and mortality of infections due to immune dysfunction, micro- and macro-angiopathies, and neuropathy, which lead to increased numbers of interventions in this population.^8^ Moreover, the development of compartment syndrome as a result of the initial infection with concomitant soft tissue necrosis may have served as a nidus for infection exacerbation and rapid spread, further contributing to the severity of the *P. acnes* infection.^9^

*Propionibacterium acnes*’ pathogenesis involves its ability to evade destruction by macrophages, chymotrypsin, hydrogen peroxide, and human serum factors, which can potentially be attributed to its ability to inhibit chlorination and its unique secondary cell wall structure.^10^ Similar to *Staphylococcus epidermitis*, *P. acnes* can also form a biofilm on prosthetic implants, making it especially virulent following implant-replacement surgery.^10^ Its long incubation period of up to 21 days (6 days in this case), anaerobic and aerobic growth potential, and the lack of specific growth media may contribute to the paucity of reported *P. acnes* infections in hand and wrist surgery when compared with the more well-known commensals.^2^

This case demonstrates the importance of considering *P. acnes* in any resistant and persistent postoperative hand and wrist infection. A lack of evidence-based guidelines for *P. acnes* treatment complicates efficient eradication; however, most case-based reviews recommend a similar treatment course to that demonstrated in this case, including a combined regimen of irrigation and debridement as needed and systemic antibiotics with a preference for vancomycin and clindamycin. Combination therapy with rifampin and daptomycin has also shown promise for treating *P. acnes* deep-tissue infections.^2^ In this case, although vancomycin and pipercillin-tazobactam resistance were never formally demonstrated by the microbiology laboratory, some plausible explanations for resistance include bacterial wall peptidoglycan alteration from D-alanyl-D-alanine to D-alanyl-D-lactate- conformation preventing vancomycin binding, and extended-spectrum β-lactamase production for pipercillin-tazobactam resistance. In contrast, carbapenems are effective as a last resort for antibiotic-resistant species because of their unique carbapenem–β-lactam molecular structure, which confers resistance against extended β-lactamases. Considering the destructive potential of *P. acnes* deep-tissue infections, as demonstrated in this case, we recommend the creation of formal, evidence-based guidelines regarding the optimal treatment for deep-tissue *P. acnes* infections to prevent similar instances to this case moving forward.
